# Impact of Inclusive Leadership on Innovation Performance During Coronavirus Disease 2019 Outbreak: Mediating Role of Employee Innovation Behavior and Moderating Role of Psychological Empowerment

**DOI:** 10.3389/fpsyg.2022.811330

**Published:** 2022-08-18

**Authors:** Shuchi Gupta, Nishad Nawaz, Abhishek Tripathi, Shafaq Arif Chaudhry, Khushbu Agrawal

**Affiliations:** ^1^Accounting Department, University of Ha’il, Ha’il, Saudi Arabia; ^2^Department of Business Management, College of Business Administration, Kingdom University, Riffa, Bahrain; ^3^Department of Management and Information Systems, College of Business Administration, University of Ha’il, Ha’il, Saudi Arabia; ^4^Lahore Business School, University of Lahore, Lahore, Pakistan; ^5^Pacific Institute of Management, Pacific University, Udaipur, India

**Keywords:** inclusive leadership, employees’ innovation behavior, innovation performance, psychological empowerment, COVID-19

## Abstract

This study investigates the effect of inclusive leadership on innovation performance with a mediating role of employee innovation behavior and the moderating role of psychological empowerment (PE). Supervisors and employees of Saudi manufacturing firms are the participants of this study. This study used a quantitative research technique with a cross-sectional approach and a self-administrative survey questionnaire to collect the data. The data were analyzed by using the Smart PLS 3 software. The results depict that inclusive leadership has a significant positive impact on the firm’s innovation performance. Employees’ innovation behavior has a significant mediating effect on the association of inclusive leadership and innovation performance. Findings revealed that PE has an important moderating role in the association of inclusive leadership and innovation performance. The findings of this study contribute to the body of knowledge by finding that inclusive leadership has a significant effect on the firm’s innovative performance and PE is crucial to enhance innovation performance.

## Introduction and Background of the Study

In 2020, the explosion of coronavirus disease 2019 (COVID-19) substantially changed the working conditions and ways of people. Uncertainty and ambiguity associated with the unprecedented pandemic resulted in various organizational challenges, including efficiency and consistency of employees regarding their job engagements during COVID-19. Active contribution of employees is equally significant in contextualizing the changing circumstances, enhancing productivity, and dealing with uncertainty. More specifically, the inclusive style of leadership and innovative attitude of employees potentially support organizations in making effective and responsive decisions. In addition, innovative behavior effectively communicates valuable information within an organization that enhances the resistance capacity of an organization during a crisis (Wang et al., 2010).

In the era of globalization, the intensity of competition among competitors is increasing. In organizations, innovation has a greater significance at present which directly decides its existence and end (Gumusluoglu and Ilsev, 2009). In addition, in response to changing demands of customers in the contemporary competitive culture, organizations must explore and promote innovative ways (Battistelli et al., 2013; Javed et al., 2019a). Literature recognized that research and development are not merely associated with the researchers but are also concerned with the other employees. For the sustainable success of organizations, areas of innovation must be open for employees with their particular roles (Dorenbosch et al., 2005; Imran and Anis-ul-Haque, 2011; Qu et al., 2017; Schermuly et al., 2017). Organizational innovation is not different from employee innovation, which is a vital element in the development of an entity. Many researchers are focusing on finding the methods of improving performance of the employees. Many factors, including intrinsic motivation and employees’ character, are significant which influence innovative performance of the employees (Shalley et al., 2009; Grant and Berry, 2011). Besides these personal factors of an individual, leadership style also influences the innovative performance of an individual (Scott and Bruce, 1994b). In recent years, a new type of leadership has been predicted, which is known as inclusive leadership. Inclusive leadership arose in response to employees’ diversity of values, personalities, and working mechanisms. In inclusive leadership, leaders deal with the employees in different manners to fulfill various needs of employees by developing a supportive employee environment and providing the foundation for the innovative performance of employees.

Moreover, an inclusive leadership style accelerates the competence and self-efficacy of employees while performing their tasks by allowing them to contribute to decision-making (Javed et al., 2019a). Besides, in this kind of leadership, employees can practice higher independence of decision-making in their undertakings by delegating power to employees (Nishii and Mayer, 2009). Empirical evidence of recent studies also supports the positive influence of diverse leadership styles in understanding the needs of employees in the situation of uncertainty, i.e., COVID-19 (Lee et al., 2020).

To the best of the researcher’s knowledge, this is a pioneer study examining the relationship of inclusive leadership and employees’ innovative performance in the COVID-19 outbreak. In the process of innovation, employees use their capabilities and exhibit differentiating behavior where innovative ideas are generated, implemented, and promoted (Ramamoorthy et al., 2005). As a result, an innovative work environment is developed (De Jong and Den Hartog, 2010) to effectively deal with the rapidly changing climate (Javed et al., 2019b). In promoting innovative work behavior (IWB) of employees, leadership is recognized as a significant factor (Scott and Bruce, 1994b; To et al., 2015; Choi S. B. et al., 2016). Leadership role at the workplace is considered as a critical factor of change and innovation within an enterprise (De Jong and Den Hartog, 2008, 2010; Amabile and Kramer, 2012).

The traditional “leader-centric” approach (Epitropaki and Martin, 2005; Lapierre et al., 2006) focuses on the behavior and attitude of leader and assumes the character of followers to be unchanged (Drath et al., 2010; Javed et al., 2019b). In contrast, inclusive leadership pays attention to the characteristics of leaders and the behavior and attitude of employees, and their affiliation toward their leader (Maslyn et al., 2017). Therefore, relational leadership theory inspires employees to handle the complexity of IWB with cooperation and support (Burke et al., 2003; Schermuly et al., 2013; Javed et al., 2019b). In addition, IWB is a complex and extraordinary behavior of employees where they communicate innovative ideas, avoid traditional mechanisms, and challenge the *status quo* by disagreeing with the opinion of their managers (Janssen, 2000; Kessel et al., 2012). Accordingly, it is perceived that many of the innovative ideas remain flop (Mathisen et al., 2012) which ultimately influence innovation performance. Rahman et al. (2015) concluded that employees’ opinion for new developments is not accepted because it is anticipated as deviant behavior in the work environment. In return, innovative employees are perceived as disturbance creators by their leaders (Kaptein, 2011). Hence, these employees face penalties that may include demotion or termination as a reward for their innovative thinking (Detert and Treviño, 2010). Therefore, for managing the complexity of the IWB process, the psychological support of employees is essential to encourage their participation in IWB (Wagner et al., 2010; Afsar and Badir, 2016). As a result of psychological empowerment (PE), individuals enjoy a sense of independence, purpose, capabilities, and response while practicing IWB (Montani et al., 2014; Orth and Volmer, 2017; Ertürk and Albayrak, 2019). Furthermore, intrinsic motivation positively influences performance regarding innovation (Ryan and Deci, 2000a).

Cognitive evaluation theory recommends that intrinsic motivation or PE allows employees to enjoy the sense of independence, purpose, capabilities, and response while practicing innovative behavior of the employees (Deci et al., 1975, 1989; Ryan and Deci, 2000b; Deci and Ryan, 2013) that ultimately influence their innovative performance (Chen and Hou, 2016; Javed et al., 2017). According to the CET theory, external factors are evaluated by employees to behave in a particular way (Deci and Ryan, 2000; Kent, 2014). Likewise, concerning the innovation, external context is evaluated by employees to find help for their IWB. If they remain successful in finding a supportive environment, their motivation toward IWB is enhanced (Yidong and Xinxin, 2013). Accordingly, PE significantly plays the moderating role in the relation of inclusive leadership and innovation performance that is less investigated in the previous research. In recent times, Javed et al. (2018) recommended that more research is needed for a detailed evaluation of the PE role related to inclusive leadership and innovation performance. In line with this, this research attempts to evaluate the effect of inclusive leadership on innovation performance of employees with mediating role of PE and moderating role of innovation behavior of employees.

The conceptual framework for this research was established from previous findings and theoretical gaps were discovered in the literature. Theoretically speaking, the value of this research is that it has established in explaining the direct relationships of inclusive leadership on innovation performance during COVID-19. By investigating the mediation effect of employee innovation behavior in the relationship between total inclusive leadership and innovation performance during COVID-19, the research has supported the past theoretical background. In brief, the results provide a new direction for the studies on small and mid-size enterprises (SME) performance and its predictors in Kingdom of Saudi Arabia (KSA)-based manufacturing firms. This study narrows the gap in the management literature regarding the role of a mediator and a moderator. As discussed in previous studies, examining the indirect relationship has been widely accepted as an investigative approach.

## Literature Review

### Employee Innovation Performance

Employee innovation performance is viewed as a process based on certain steps (Yi, 2008), by considering it as a process. There are five steps included in the process, namely, willingness, action, suggestion, achievement, and the communication of innovative ideas (Song et al., 2015). In addition, Janssen et al. (2011) recommended that innovation performance of employees is an advanced idea that boosts the performance of an entity. Yuanyuan (2013) considered employee innovation performance in two parts that are innovation action and innovation influence. Innovative action refers to the new ideas and programs that employees have. However, the innovation effect is associated with the innovation achievement and implication of outcomes. This study describes employee innovation performance by the model of Han et al. (2011).

### Inclusive Leadership

UN Millennium Development Goals include the concept of Inclusion and it is an old feature of Chinese civilization (Fang et al., 2019). Inclusiveness is regarded as traditional virtue in the Chinese population. Phrases such as “All rivers run into the sea” and “Wide hearts embrace all” carry the concept of inclusiveness in their meaning. Initially, the concept of inclusive leadership was evaluated in Western education, where it was suggested that individuals belonging to various races and capacities should have inclusive education opportunities. Ryan and Haslam (2007) supposed that education leadership needed an identical and collective leadership mechanism by describing inclusive leadership concerning education as the existence of a learning leader. In the subject of management, Nembhard and Edmondson (2006) first suggested inclusive leadership that incorporates the communication and behavior of leaders in inspiring their employees to make a positive contribution toward their work. Hollander (2013) focused on the perceived leadership role of employees and defined this association independently whereby having a shared vision. By considering the research of Hollander (2013) and Carmeli et al. (2010a) alleged that inclusive leadership may be evaluated from the collaboration of leaders and employees, and it is open, operative, and available while communicating with workers. In addition, Hirak et al. (2012) concluded a positive and significant association of inclusive leadership with the psychological security of subordinates while studying a large hospital. Simsek et al. (2015) also examined the concept of inclusion and recommended that two components, i.e., belonging and authenticity, should be incorporated in it. Accordingly, inclusion is defined by the researchers as the feeling of security and belonging from the team members for each other. Suk considered inclusive leadership as an open, operative, and easy to learn leadership method that positively influences performance of the employees (Choi S. L. et al., 2016).

Later on, Chinese researchers focused on inclusive leadership, and, at present, a number of studies are ongoing on this subject. Fang et al. (2014) determined that inclusive leaders focus more keenly on the association of leaders and employees by combining the features of transformational and transactional leadership and taking benefit of the authentic style leadership and shared leadership. Moreover, Guan and Liu (2016) highlighted that inclusive kind of leadership focuses on the people-oriented principals, pays attention to the equal treatment toward various attitudes of subordinates, and recommends the role of managerial consistency, and the efforts of leaders are presented as a role model. Furthermore, Liu et al. (2017) concluded that inclusive leadership follows people-oriented principles, supports differences of opinion among individuals, pays significance to the interaction of leadership and employees, and considers contributions and opinions of employees prominently. In this research, the concept of inclusive leadership is integrated with the traditional Chinese cultural concept of “inclusiveness.” In the West, the idea of inclusiveness is mainly based on the conceptions of democracy and justice. Inclusiveness in the Chinese traditions is concerned more about the “tolerance and greatness” of moral values and mind. Inclusive leaders integrated with Chinese culture pay more focus on equal distribution and fair opportunity and are regarded as a new kind of democratic leadership. It is in line with the higher psychological perceptions and associated needs of employees in the current era. In inclusive leadership, leaders treat employees with more gratitude, admiration, and acceptance (Sharifirad, 2013). They pay value to the contribution and thoughts of employees and encourage their performance in the workplace (Kang et al., 2015). Meanwhile, inclusive managers focus on training employees, giving them fair treatment, and taking the business to the achievement (Fang et al., 2021).

In the interaction of leaders and subordinates, inclusive leadership can help (Nishii and Mayer, 2009). However, relational leadership refers to the interaction of leaders with subordinates (Rawat et al., 2020) that is also responsible for performance. It is an example of fairness and openness (Wang and Zhu, 2011). Nembhard and Edmondson (2006), in their model, categorized the inclusive leadership scale into the dimensions of “invitation” of the leader and “appreciation” of the followers. In the inclusive leadership scale of Hollander (2013), “support-recognition,” “communication-action-fairness,” and “self-interest-disrespect” were included in the comprehensive evaluation. By considering literature and empirical research, the concept of encouragement, recognition, and inclusiveness is included in the advanced practices of leadership. First, leaders are perceived to pay value to the opinion of subordinates, expressively consider encouragement of employees, and recognize achievements of employees. Second, leaders are supposed to deal with the employees in fair manners. Accordingly, leaders may deal with the employees in fair and just manners by respecting their proposals and letting them to do more in order to gain more. Finally, leaders are supposed to understand employees in rational manners by tolerating their failures and mistakes. Leaders, on mistakes, can understand employees rationally and tolerate them.

### Employees’ Innovative Behavior

The innovative behavior concept began in the decade of 1970s. There are three levels of innovative behaviors, namely, organization, team, and individual. Concerning this research, the individual innovative behavior of organizational workers is included in the examination. In addition, Amabile (1988a) described innovation as the creativity of employees that can be a valuable thought or action, which ultimately encourages and enables entities to continue, flourish, and grow well in the intensely competitive environment. Amabile et al. (2018) added that the ideas produced by employees of innovation might potentially be or have already been applied. Zhou and George (2001) recommended that the innovative behavior of an employee is not merely associated with the generation of new ideas but also includes promotion and application of an innovative idea. Woodman et al. (1993) believed that innovative behavior comprises the process of producing innovative thoughts and their effective application. Scott and Bruce (1994a) divided innovation into three stages, i.e., recognizing problems and finding solutions for problems, looking for backing for their ideas, and establishing innovative principles that may be communicated, mass-produced, and applied at an enormous scope. Moreover, De Jong and Den Hartog (2010) divided innovative behaviors of individual employees into five steps, i.e., discovering opportunities, producing ideas, establishing surveys, supporting, and implementing. In China, researchers also initiated studies on this topic. Jiang et al. (2015) and Yang (2011) explained innovative behavior of employees in relation to the generation and application of innovative and applicable mechanisms, while employees are undertaking associated activities in the organization. Likewise, Li et al. (2017) indicated that innovative behavior is concerned with the process where employees highlight issues, provide innovative ideas, and communicate and apply these innovative ideas in the whole period of an enterprise. Based on the questionnaires developed by Scott and Bruce, this research divides employees’ innovative behavior into two dimensions, namely, “innovation outcomes” and “innovative thinking.” Innovative outcomes refer to the impacts of new idea application in organizational operations. In contrast, innovative thinking is concerned with generating innovative ideas by employees in the business process.

### Hypotheses Development

#### Inclusive Leadership and Employee Innovation Performance

Innovation performance of employees is incredibly significant for an organization, and many factors influence it. From those factors, researchers found that leadership style more significantly decides the performance of an innovative team (Eisenbeiss et al., 2008). Inclusive leadership style is shaped by openness, tolerance, and support. The organizational support concept states that inclusive leaders encourage employees to practice positive behavior at the workplace in business (Choi et al., 2015). Literature witnessed that where a leader practices a more supportive attitude toward the employees’ innovation, they accomplish better innovative outcomes (Madjar et al., 2011). In addition, the study found that the inclusive style of leadership indirectly influences the innovative performance of teams by acknowledging and promoting suggestions of team members (Xiang et al., 2017; Yan et al., 2020).

In contemporary organizations, developing an innovative attitude is one of the most critical leadership functions (Zhu et al., 2020). Leadership style is significantly concerned with the innovative ability of the employees (Lee et al., 2011). Illustratively, a leader who is confident with the employees can express creative ideas more appropriately as an innovative attitude (Pundt, 2015). Furthermore, leaders with the transformational leadership style remain more successful in inspiring employees to innovate by incorporating intelligence and encouragement (Yang et al., 2020). Inclusive leadership style also has a positive influence from the perspective of Chinese tradition similar to other beneficial leadership styles. Innovation performance of employees increases while they are getting more engagement at higher positions because they consider that leaders are acknowledging their performance (Alosani et al., 2021; Raoof et al., 2021). Leaders’ support and encouragement also has an impact on the innovative behavior of individuals. They are more productive and innovative when they have backing from their leaders (George and Zhou, 2007). Furthermore, Javed et al. (2019a) added the concept of “fault-tolerant” in inclusive leadership style in the Chinese context and concluded its positive influence on employee’s self-efficacy of employees. Additionally, Liu et al. (2017) determined the positive influence of inclusive leadership positively concerning mental models of teams where the reflection of teams plays the role of a moderator. Liu et al. (2015) more comprehensively found a positive and significant link between inclusive leadership and the creativity of employees. Jin et al. (2017) recommended that a higher degree of inclusiveness in employees’ minds has more probability of performance improvement. Escribá-Carda et al. (2017) theoretically defined inclusive leadership as a set of positive behaviors of a leader that support team members and develop the feel of belonging to team members by maintaining their uniqueness within the group. Consequently, inclusive leaders more positively perceive employees and tolerate their failures that develop a sense of support and encouragement for employees resulting in additional innovative ideas (Wang and Rode, 2010). In the new era, generally, employees depict more creative ideas but have opinions constructed with a traditional leadership approach; this kind of inclusiveness comprises inspiration, and tolerance is highly effective. Therefore, the following hypotheses based on an extensive literature review are proposed:

**Hypothesis 1:** There is a significant association between inclusive leadership and employees’ innovation performance in the COVID-19 outbreak.

**Hypothesis 2:** There is a significant association between inclusive leadership and employees’ innovation behavior in the COVID-19 outbreak.

#### The Mediating Role of Employee Innovation Behavior

A series of activities included in the innovative behavior comprises of generation of an idea, its promotion, and recognition of innovative technologies, operations, methods, and offerings (Janssen and Van Yperen, 2004; Yuan and Woodman, 2010). Innovation behavior of employees is more concerned about the process of innovation instead of innovative results or innovative products (Montag et al., 2012; Shin et al., 2017) that is different from the perception of creativity (Shin et al., 2017). This study developed a theoretical framework based on the research by Shin et al. (2017) where they incorporated literature on innovative behavior in general and considered literature regarding creativity. In the past, numerous kinds of leaderships are evaluated to assess their impact on the innovative behavior of employees in business (Mumford et al., 2002). In this way, Amabile (1988b) recommended that autonomy, encouragement from managers, and organizational backing are closely associated with the workers’ innovation. Concerning the theory, inclusive leaders in multiple ways can influence the innovative behavior of employees. Primarily, inclusive leaders may strengthen employees to contribute to the innovative process (Atwater and Carmeli, 2009). Moreover, Conger et al. (1997) treated inclusion as a concept of intrinsic motivation and a process that improves the internal perception of employees in an organization. The higher degree of motivation results in the greater involvement of employees in practicing innovative attitudes (Shin and Zhou, 2003; Atwater and Carmeli, 2009). After that, based on the story of organizational support (Shin and Zhou, 2003), the working outcomes of employees depend upon organizational support. In inclusive leadership, leaders can provide resources of knowledge, time, and support that are needed for an innovative attitude (Reiter-Palmon and Illies, 2004). Hence, the inclusiveness of leaders is associated with regarding and encouraging various opinions of different members of the interacting team (Mitchell et al., 2015; Randel et al., 2018). Where employees support their leaders, they feel more independence and autonomy while practicing innovative behavior (Foss et al., 2013).

In the same way, Boren (1994) asserted that inclusion is primarily based upon the trust of employees. Furthermore, Randel et al. (2018) determined that inclusive behavior of leaders potentially supports employees in developing a perception of belongingness where leaders support team members, they are equally treated, and are included in making a decision. In maintaining the uniqueness of employees, leaders encourage diverse viewpoints by supporting members to contribute fully inside an organization (Randel et al., 2018). In addition, inclusive leaders may play the role of role models in innovative behavior processes (Jaussi and Dionne, 2003). Carmeli et al. (2010b) asserted that the inclusiveness of leaders has a positive impact on the engagement of employees in the work of quality improvement. Furthermore, Hirak et al. (2012) have the opinion that those inclusive leaders establish a unique relationship that is practiced through harmony and frankness in communication and accessibility (Carmeli et al., 2010b). With the help of appropriate inclusive behavior, such an environment is developed by leaders where employees take greater responsibility (Borman and Motowidlo, 1993), enjoy more autonomy while making a decision, and have greater access to the feedback and information combined with the encouragement and support (Arnold et al., 2000). Participation of employees in innovative tasks is assisted by openness and accessibility (Carmeli et al., 2010b). In literature, innovative behavior is occasionally termed as “discretionary behavior” (Janssen, 2000). An exceptional feature of inclusive leadership is to reshape followers’ perceptions and enhance their participation in innovative behavior (Randel et al., 2018). Therefore, the following hypotheses based on organizational support theory (Riggle et al., 2009) are proposed:

**Hypothesis 3:** There is a significant association between employees’ innovation behavior and employee innovation performance in the COVID-19 outbreak.

**Hypothesis 4:** Employees’ innovation behavior significantly mediates the relationship between inclusive leadership and employee innovation performance.

#### The Moderating Role of Psychological Empowerment

Team empowerment is concerned with the enhanced intrinsic innovation toward a task established based on four dimensions of employee regarding his or her work position that includes the meaning, capability, self-determination, and outcome (Spreitzer, 1995). Spreitzer (1995), based on the above definition, recommended four dimensions of PE: purpose, capability, self-determination, and outcome. First, meanings refer to the value or importance that individuals practice toward their task while performing it. Second, capacity or competence is concerned with the qualification or ability that an employee needs to accomplish the allocated task. Third, self-determination is the degree of independence and autonomy that an employee perceives while performing the task. Finally, outcome refers to the expectation of employees that their accomplished task will contribute a positive change in the objectives of their organization (Spreitzer et al., 1997; Seibert et al., 2011; Jose and Mampilly, 2014). The concept of empowerment is primarily based upon the idea of decentralization, where the authority of decision-making is entrusted to the employees at lower levels to achieve the best results (Barton and Barton, 2011; Pardo-del-Val et al., 2012). Hence, researchers in this study propose that PE has a mediating role in the relationship of inclusive leadership and the innovative performance of the employee.

Inclusive leaders employ numerous approaches for enhancing the innovative performance of subordinates. Inclusive leadership firstly focuses on the various integral principles of exclusivity and belongingness that promote respect and the individual position of an employee (Randel et al., 2018) and further develop meaning for employees at work. In addition, individuals associated with inclusive leaders learn key competencies needed to perform a task appropriately from the ongoing process of leadership (Carmeli et al., 2010a; Choi, 2017; Shore et al., 2018; Zafar et al., 2021). Moreover, leaders in inclusive leadership vest independent authority to the individuals to decide activities of their task with higher self-determination and confidence level (Shore et al., 2018). In the end, with the accessibility attribute of inclusive leadership, timely feedback is received by employees (Carmeli et al., 2010b) that enables them to evaluate the impact of their efforts on the performance. In the literature, some of the studies recommend that inclusive leadership enhances the performance of employees in an enterprise. Accordingly, Javed et al. (2019a) found the association between inclusive leadership and PE while testing a sample of cargo and information technology (IT) employees in the context of Canada and the United Kingdom correspondingly. Similarly, the results of another study concluded that inclusive leadership promotes the PE of employees and yields required results (Tuuli and Rowlinson, 2009). A positive correlation between PE and the performance of employees is found in the studies (Ke and Zhang, 2008). Numerous researchers concluded that PE was positively associated with the success of a task (Barrett et al., 2003; Rowlinson and Cheung, 2008; Nauman et al., 2010; Tuuli et al., 2015; Prihatiningsih, 2016; Hu et al., 2018). Therefore, following hypothesis based on the above-debated literature is proposed.

**Hypothesis 5:** Psychological empowerment significantly moderates the relationship of inclusive leadership with employee innovation performance.

### Research Methodology

Methodology for any research is selected based on the problem of research or objectives of the research (Sabir et al., 2019) and proper methods are obligatory for the preciseness of research findings. The cross-sectional method with a quantitative research approach was selected to investigate the problem of this research study. A self-administrative questionnaire was used to collect the data. The questionnaire technique for data collection is the best because it makes it convenient to collect the data at a reasonable cost and time (Sekaran and Bougie, 2003). This study used multistage sampling process to collect the data.

The following service industries were selected, namely, banking, real estate, healthcare, telecommunications, and insurance. Then, 25 companies were selected from the above sectors and the data were collected from the employees of selected companies. Scales items for all variables of this study (see Appendix-1) were adapted from previous studies. The nine items for inclusive leadership were adapted from the study of Choi et al. (2017), four-item scale for innovation performance was adapted from the study by Mumtaz and Parahoo (2019), and nine-item scale of employee innovation behavior was adapted from Luthans et al. (2007). The 11 items of the PE scale proposed by Spreitzer (1995) were employed in this study. This study is conducted on the employees and supervisors of Saudi manufacturing firms.

### Sample Size

The study follows the instructions of Comrey and Lee (1992) inferential statistics in the selection of sample size to collect the data. Comrey and Lee (1992) argued that a sample size of less than 50 respondents is a weaker sample, 100 is considered a weak sample, 200 is assumed an adequate sample, and 300 is assumed a good sample. Therefore, the current study chose a sample size of 300 that is considered a good sample. Missing data were treated by using the “pair-wise-deletion” and filling in the missing value with estimation is the recommended option (Singh, 2007). Hair et al. (2013) recommended that the missing values should be replaced with the mean value if these are less than 5%. The missing value ranged from 0.80 to 2.13%; thus, these were substituted by SPSS.

## Analysis and Results

**FIGURE 1:**
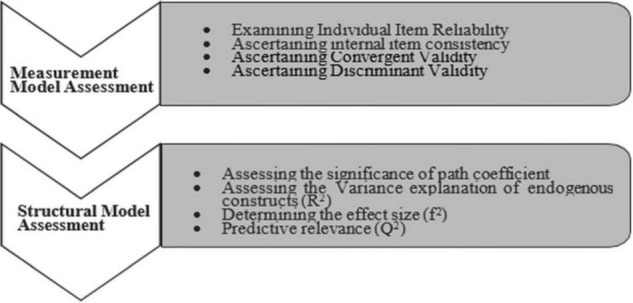
A two-step process of PLS path model assessment. Source: Henseler et al. (2009).

The data were analyzed by using the statistical software, Smart PLS 3. Two-step approach by Henseler et al. (2009) was applied for the analysis of data. Table 1 shows the response rate and the Figure 1 shows the two-step PLS-SEM process.

**TABLE 1:** Response from respondents.

Response	Frequency/Rate
Total questionnaires distributed	300
Total questionnaires returned	231
Total useable questionnaires	213
Total questionnaires excluded	18
Total response rate after data entry	71%

### Measurement Model Assessment

**FIGURE 2:**
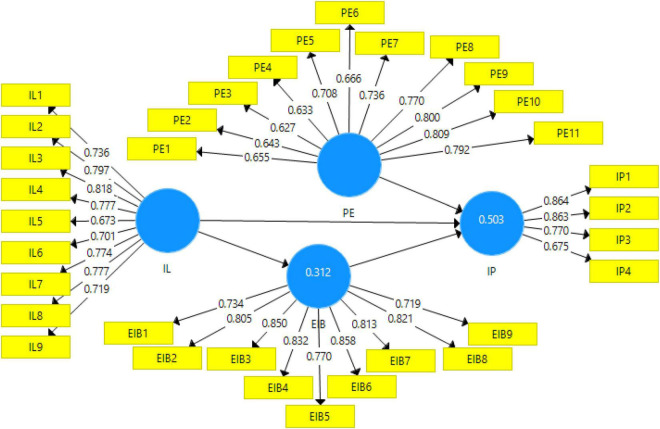
Measurement model assessment.

Before testing the hypotheses of the study, measurement model was assessed to confirm the discriminant and convergent validities (Ringle et al., 2015). Convergent validity is assessed from the values of Cronbach’s alpha, composite reliability, and average variance extracted (AVE). The value of AVE should be equal to or greater than 0.50 and the value of CR should be equal to or above 0.60 to establish the convergent validity (Bagozzi and Yi, 1988). The statistical results show that this study had established convergent validity. The results of the measurement model are shown in Figure 2 and Table 2.

**TABLE 2:** Internal consistency, convergent validity, composite reliability, and AVE.

Construct	Indicators	Loadings	Cronbach’s alpha	Composite reliability	AVE
Innovation performance (IP)	IP1	0.864	0.808	0.873	0.635
	IP2	0.863			
	IP3	0.770			
	IP4	0.675			
Inclusive leadership (IL)	IL1	0.736	0.905	0.922	0.568
	IL2	0.797			
	IL3	0.818			
	IL4	0.777			
	IL5	0.673			
	IL6	0.701			
	IL7	0.774			
	IL8	0.777			
	IL9	0.719			
Employees innovation behavior (EIB)	EIB1	0.734	0.930	0.942	0.643
	EIB2	0.805			
	EIB3	0.850			
	EIB4	0.832			
	EIB5	0.770			
	EIB6	0.858			
	EIB7	0.813			
	EIB8	0.821			
	EIB9	0.719			
Psychological empowerment (PE)	PE1	0.655	0.905	0.920	0.512
	PE2	0.809			
	PE3	0.792			
	PE4	0.643			
	PE5	0.627			
	PE6	0.633			
	PE7	0.708			
	PE8	0.666			
	PE9	0.736			
	PE10	0.770			
	PE11	0.800			

*Authors’ estimates based on survey data.*

According to the study by Fornell and Lacker (1981), discriminant validity is confirmed if the value of square root of a particular variable of AVE is greater than the correlation of that particular variable with other variables of the model. Table 3 represents the square root of AVE.

**TABLE 3:** Fornell–Larcker criterion.

	EIB	IL	IP	PE
EIB	0.802			
IL	0.561	0.754		
IP	0.541	0.443	0.797	
PE	0.614	0.631	0.699	0.716

*Authors’ estimates based on survey data.*

Heterotrait-Monotrait (HTMT) ratio is an alternative method to examine the discriminant validity. According to the study by Kline (2011), HTMT ratio should be less than 0.85 to establish the discriminant validity. Table 4 summarizes the value of HTMT ratios.

**TABLE 4:** Heterotrait-Monotrait ratio (HTMT).

	EIB	IL	IP	PE
EIB				
IL	0.595			
IP	0.603	0.502		
PE	0.672	0.714	0.788	

*IP, innovation performance; IL, inclusive leadership; EIB, employees’ innovation behavior; PE, psychological empowerment.*

### Structural Model Assessment

**FIGURE 3:**
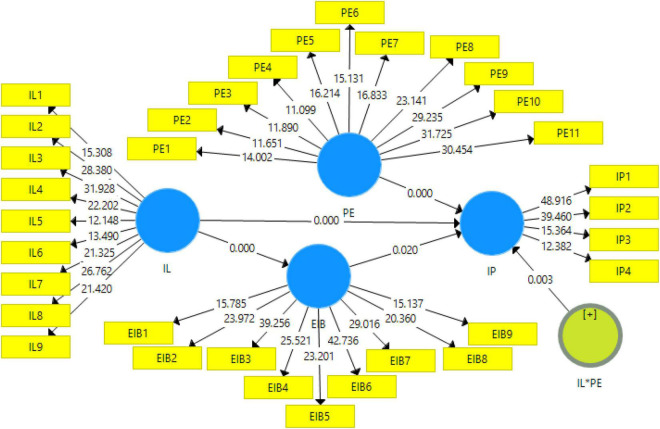
Structural model assessment.

The PLS was used for SEM estimation and testing the hypotheses of the study. The graphical representation of structural model assessment is given in Figure 3. The bootstrapping procedure was applied to test the effect and hypotheses of the study. Findings of SEM presented in Table 5 show that the results indicated that Inclusive Leadership has a significant and positive effect on Innovation Performance (β = 0.266, *t* = 2.860); hence, H_1_ is accepted. Furthermore, findings revealed that Inclusive Leadership also has a significant and positive relationship with Employees Innovation Behavior (β = 0.561, *t* = 9.660) and H_2_ is accepted. Employees’ Innovation Behavior is also significantly and positively related to Innovation Performance and H_3_ is accepted. This study adopts the method developed by Hayes (2009) to test the mediation effect and apply the bootstrapping procedure of PLS-SEM. Results revealed that Employees Innovation Behavior significantly and fully mediates the relationship of Inclusive Leadership with Innovation Performance (β = 0.118, *t* = 2.157) and H_4_ is accepted. Results show that PE has a significant and positive effect on the relationship of Inclusive Leadership with Innovation Performance (β = 0.189, *t* = 2.864). Therefore, H5 is accepted on the statistical ground.

**TABLE 5:** Structural model assessment.

Hypotheses	Relationship	Beta	SD	*T* statistics	*P*-values
H_1_	IL → IP	0.266	0.093	2.860	0.000
H_2_	IL → EIB	0.561	0.058	9.660	0.000
H_3_	EIB → IP	0.210	0.090	2.329	0.020
H_4_	IL → EIB →IP	0.118	0.055	2.157	0.031
H_5_	IL × PE → IP	0.189	0.066	2.864	0.004

*Authors’ estimates based on survey data.*

## Discussion and Conclusion

This study aimed to investigate the role of inclusive leadership on innovation performance with a mediating role of employee innovation behavior and a moderating role of PE. This study found that inclusive leadership significantly affects innovation performance, especially in the COVID-19 outbreak. Employees feel more care and value when they perceive that their leaders show more inclusiveness regarding their new processes, technologies, and ideas, and therefore, the innovation performance of employees is positively affected. This result is in line with organizational support theory and the study of Qi et al. (2019). Furthermore, the results of this study revealed that employees’ innovation behavior has a significant mediation effect between the relationship of inclusive leadership and innovation performance. Inclusive leadership is a form of leadership that maintains a direct relationship with the employees and allows them to take part in decision-making that develops participative behavior among employees. This creates IWB among employees that ultimately boosts innovation performance (Choi et al., 2017; Javed et al., 2019a). Results also depict that PE significantly moderates the relationship of inclusive leadership with innovation performance. PE enhances the effect of inclusive leadership on innovation performance in many ways. For example, inclusive leadership engages employees in a creative and constructive discussion that enhances an intellect of meaning at work and employees learn essential capabilities of proficiently performing an assigned role (De Hoogh and Den Hartog, 2008). This study contributes to the body of literature on inclusive leadership in many ways. The direct association between inclusive leadership and innovation performance has already been recognized. However, this study has investigated the indirect association between inclusive leadership and innovation performance *via* the mediation of employees’ innovation behavior, especially in the era of the COVID-19 outbreak. Moreover, this study makes a contribution by confirming the moderating role of PE on the relationship of inclusive leadership with innovation performance.

### Theoretical Implications

This study address three unanswered questions of the leadership literature by incorporating the Leadership-Member Exchange theory. First, the association between IL and innovative performance was evaluated and it was found that IL significantly increases innovative performance that is a new contribution toward the relevant stream of literature. Second, mediating roles of employee innovation behavior were examined in the association of IL and innovative performance and found the significant mediating role of employee innovation behavior in the framework. Third, the moderating role of PE was examined concerning IL and innovative performance. By using this approach, new dimensions of thinking are highlighted in the literature that is how IL is contributing more toward the innovative performance.

### Practical Implications

Practically, this research has numerous implications. Primarily, organizations need to appoint managers who practice inclusive behavior for innovative performance. In the contenders of managers, judgment is possible based on IL attributes while selecting managers. In addition, training managers is important for promoting a culture of respect for all employees, praising the role of each worker, and paying attention to the different opinions of employees. Moreover, managers are required to act as role models and they should share resources, power of decision making, and should offer constructive and timely feedback for the better psychologically empowered experience of the employees. By providing training by keeping in view the above important considerations of IL, firms can encourage IL in the managers who ultimately will improve organizational performance.

### Limitations

The present study has some limitations that are important to consider before concluding. First, because of practical restrictions in evolving a probabilistic sampling frame, this study used a convenience sampling; due to this limitation, the representative sample was adopted very carefully. Moreover, sample size of the study is not too large and imposes a limitation on the generalizability of the results. For these reasons, future researchers are required to consider a larger sample size while conducting these kinds of studies. In addition, only PE is incorporated as a mediating variable in the association of IL and innovative performance. In the future, researchers can consider additional mediating variables while evaluating this association.

## Data Availability Statement

The data supporting the conclusions of this article will be made available by the authors, without undue reservation.

## Ethics Statement

Ethical review and approval was not required for the study on human participants in accordance with the local legislation and institutional requirements. Written informed consent for participation was not required for this study in accordance with the national legislation and the institutional requirements.

## Author Contributions

SG, NN, and SA developed the main conceptual idea and developed the theoretical framework. NN, AT, and KA collected the data and performed the numerical calculations for the data. SG, AT, and SA wrote the manuscript. All authors listed have made a substantial, direct, and intellectual contribution to the work, and approved it for publication.

## Conflict of Interest

The authors declare that the research was conducted in the absence of any commercial or financial relationships that could be construed as a potential conflict of interest.

## Publisher’s Note

All claims expressed in this article are solely those of the authors and do not necessarily represent those of their affiliated organizations, or those of the publisher, the editors and the reviewers. Any product that may be evaluated in this article, or claim that may be made by its manufacturer, is not guaranteed or endorsed by the publisher.

## Appendix

**Appendix 1:** Measured items.

Variable	Items	Items in details
**Inclusive leadership**	IL1	The manager is open to hearing new ideas
	IL2	The manager is attentive to new opportunities to improve work processes
	IL3	The manager is open to discussing the desired goals and new ways to achieve them
	IL4	The manager is available for consultation on problems
	IL5	The manager is an ongoing “presence” in this term-someone who is readily available
	IL6	The manager is available for professional questions I would like to confirm with him/her
	IL7	The manager is ready to listen to my requests
	IL8	The manager encourages me to access him/her on emerging issues
	IL9	The manager is accessible for discussing emerging problems
**Employee innovation behavior**	EIB1	I search out new working methods and techniques
	EIB2	I search out new instruments for working
	EIB3	I generate original solutions for problems
	EIB4	I make important organizational members enthusiastic for innovative ideas
	EIB5	I transform innovative ideas into useful applications
	EIB6	I introduce innovative ideas into the work environment in a systematic way
	EIB7	I evaluate the utility of innovative ideas
	EIB8	I perform a task by using innovative methods
	EIB9	Applications of innovative ways problem solving give me pleasure
**Innovation performance (IP)**	IP1	Coming up with new ideas
	IP2	Working to implement new ideas
	IP3	Finding improved ways to do things
	IP4	Creating better processes and routines
**Psychological empowerment (PE)**	PE1	The work I do is very important for me
	PE2	I am self-assured about my capabilities to perform my work activities
	PE3	The work I do is meaningful to me
	PE4	I am confident about my ability to do my job
	PE5	My job activities are personally meaningful to me
	PE6	I have mastered the skills necessary for my job
	PE7	Can decide on my own how to go about doing my work
	PE8	I have considerable opportunity for independence and freedom in how I do my job
	PE9	My impact on what happens in my department is large
	PE10	I have a great deal of control over what happens in my department
	PE11	Have significant influences on what happens in my department

## References

[B1] Afsar B. Badir Y. F. (2016). Person–organization fit, perceived organizational support, and organizational citizenship behavior: the role of job embeddedness. *J. Hum. Resour. Hosp. Tour.* 15 252–278. 10.1080/15332845.2016.1147936

[B2] Alosani M. S. Al-Dhaafri H. S. Abdulla A. A. (2021). Investigating the role of HRM practices on service innovation: empirical evidence from UAE government agencies. *Manage. Res. Rev.* 44 1–24. 10.1108/mrr-03-2020-0141

[B3] Amabile T. Kramer S. (2012). How leaders kill meaning at work. *McKinsey Q.* 1 124–131.

[B4] Amabile T. M. (1988a). From individual creativity to organizational innovation.

[B5] Amabile T. M. (1988b). A model of creativity and innovation in organizations. *Res. Organ. Behav.* 10 123–167.

[B6] Amabile T. M. Collins M. A. Conti R. Phillips E. Picariello M. Ruscio J. (2018). *Creativity in Context: Update to the Social Psychology of Creativity.* London: Routledge.

[B7] Arnold J. A. Arad S. Rhoades J. A. Drasgow F. (2000). The empowering leadership questionnaire: the construction and validation of a new scale for measuring leader behaviors. *J. Organ. Behav.* 21 249–269. 10.3390/ijerph17134812 32635433 PMC7370010

[B8] Atwater L. Carmeli A. (2009). Leader–member exchange, feelings of energy, and involvement in creative work. *Leadersh. Q.* 20 264–275. 10.1016/j.leaqua.2007.07.009

[B9] Bagozzi R. P. Yi Y. (1988). On the evaluation of structural equation models. *J. Acad. Mark. Sci.* 16, 74–94. 10.1007/BF02723327

[B10] Barrett B. Marchand L. Scheder J. Plane M. B. Maberry R. Appelbaum D. (2003). Themes of holism, empowerment, access, and legitimacy define complementary, alternative, and integrative medicine in relation to conventional biomedicine. *J. Altern. Complement. Med.* 9 937–947. 10.1089/107555303771952271 14736364

[B11] Barton H. Barton L. C. (2011). Trust and psychological empowerment in the Russian work context. *Hum. Resour. Manage. Rev.* 21 201–208. 10.1016/j.hrmr.2011.02.001

[B12] Battistelli A. Montani F. Odoardi C. (2013). The impact of feedback from job and task autonomy in the relationship between dispositional resistance to change and innovative work behaviour. *Eur. J. Work Organ. Psychol.* 22 26–41. 10.1080/1359432x.2011.616653

[B13] Boren L. A. (1994). *Current Policy Development in Special Education: The Regular Education Initiative and the Inclusion Movement: A Review of the Literature.* Olympia, WA: Evergreen State College.

[B14] Borman W. C. Motowidlo S. (1993). “Expanding the criterion domain to include elements of contextual performance,” in *Personnel Selection in Organizations*, eds Schmitt N. Borman W. C. (San Francisco, CA: Jossey-Bass), 71–98.

[B15] Burke C. S. Fiore S. M. Salas E. (2003). “The role of shared cognition in enabling shared leadership and team adaptability,” in *Shared Leadership: Reframing the Hows and Whys of Leadership*, eds Bowers C. Salas E. Jentsch F. (Washington, DC: American Psychological Association), 185–212.

[B16] Carmeli A. Gelbard R. Gefen D. (2010a). The importance of innovation leadership in cultivating strategic fit and enhancing firm performance. *Leadersh. Q.* 21 339–349. 10.1016/j.leaqua.2010.03.001

[B17] Carmeli A. Reiter-Palmon R. Ziv E. (2010b). Inclusive leadership and employee involvement in creative tasks in the workplace: the mediating role of psychological safety. *Creat. Res. J.* 22 250–260. 10.1080/10400419.2010.504654

[B18] Chen A. S.-Y. Hou Y.-H. (2016). The effects of ethical leadership, voice behavior and climates for innovation on creativity: a moderated mediation examination. *Leadersh. Q.* 27 1–13. 10.1016/j.leaqua.2015.10.007

[B19] Choi G.-Y. (2017). Secondary traumatic stress and empowerment among social workers working with family violence or sexual assault survivors. *J. Soc. Work* 17 358–378. 10.1177/1468017316640194

[B20] Choi S. B. Kim K. Ullah S. E. Kang S.-W. (2016). How transformational leadership facilitates innovative behavior of Korean workers: examining mediating and moderating processes. *Pers. Rev.* 45 459–479. 10.1108/pr-03-2014-0058

[B21] Choi S. B. Tran T. B. H. Kang S.-W. (2017). Inclusive leadership and employee well-being: the mediating role of person-job fit. *J. Happiness Stud.* 18 1877–1901. 10.1007/s10902-016-9801-6

[B22] Choi S. B. Tran T. B. H. Park B. I. (2015). Inclusive leadership and work engagement: mediating roles of affective organizational commitment and creativity. *Soc. Behav. Pers. Int. J.* 43 931–943. 10.2224/sbp.2015.43.6.931

[B23] Choi S. L. Goh C. F. Adam M. B. H. Tan O. K. (2016). Transformational leadership, empowerment, and job satisfaction: the mediating role of employee empowerment. *Hum. Resour. Health* 14 1–14. 10.1186/s12960-016-0171-2 27903294 PMC5131441

[B24] Comrey A. Lee H. (1992). “Interpretation and application of factor analytic results,” in *A First Course in Factor Analysis*, eds Comrey A. L. Lee H. B. (Hillsdale, NJ: Lawrence Eribaum Associates).

[B25] Conger J. A. Kanungo R. N. Menon S. T. Mathur P. (1997). Measuring charisma: dimensionality and validity of the Conger-Kanungo scale of charismatic leadership. *Can. J. Adm. Sci.* 14 290–301. 10.1111/j.1936-4490.1997.tb00136.x

[B26] De Hoogh A. H. Den Hartog D. N. (2008). Ethical and despotic leadership, relationships with leader’s social responsibility, top management team effectiveness and subordinates’ optimism: a multi-method study. *Leadersh. Q.* 19 297–311. 10.1016/j.leaqua.2008.03.002

[B27] De Jong J. Den Hartog D. (2010). Measuring innovative work behaviour. *Creat. Innov. Manage.* 19 23–36. 10.1111/j.1467-8691.2010.00547.x

[B28] De Jong J. Den Hartog D. N. (2008). Innovative work behavior: measurement and validation. *EIM Bus. Policy Res.* 8 1–27.

[B29] Deci E. L. Cascio W. F. Krusell J. (1975). Cognitive evaluation theory and some comments on the Calder and Staw critique. *J. Pers. Soc. Psychol.* 31 81–85. 10.1037/h0076168

[B30] Deci E. L. Connell J. P. Ryan R. M. (1989). Self-determination in a work organization. *J. Appl. Psychol.* 74 580–590. 10.1186/s13104-020-05432-4 33413581 PMC7791652

[B31] Deci E. L. Ryan R. M. (2000). The” what” and” why” of goal pursuits: human needs and the self-determination of behavior. *Psychol. Inq.* 11 227–268. 10.1207/s15327965pli1104_01

[B32] Deci E. L. Ryan R. M. (2013). *Intrinsic Motivation and Self-Determination in Human Behavior.* Berlin: Springer.

[B33] Detert J. R. Treviño L. K. (2010). Speaking up to higher-ups: How supervisors and skip-level leaders influence employee voice. *Organ. Sci.* 21 249–270. 10.1287/orsc.1080.0405

[B34] Dorenbosch L. Engen M. L. V. Verhagen M. (2005). On-the-job innovation: the impact of job design and human resource management through production ownership. *Creat. Innov. Manage.* 14 129–141. 10.1111/j.1476-8691.2005.00333.x

[B35] Drath W. H. Palus C. J. McGuire J. B. (2010). “Developing interdependent leadership,” in *The Center for Creative Leadership Handbook of Leadership Development*, 3rd Edn, eds Van Velsor E. McCauley C. Ruderman M. (San Francisco, CA: JosseyBass).

[B36] Eisenbeiss S. A. Van Knippenberg D. Boerner S. (2008). Transformational leadership and team innovation: integrating team climate principles. *J. Appl. Psychol.* 93 1438–1446. 10.1037/a0012716 19025260

[B37] Epitropaki O. Martin R. (2005). From ideal to real: a longitudinal study of the role of implicit leadership theories on leader-member exchanges and employee outcomes. *J. Appl. Psychol.* 90 659–676. 10.1037/0021-9010.90.4.659 16060785

[B38] Ertürk A. Albayrak T. (2019). Empowerment and organizational identification: the mediating role of leader-member exchange and the moderating role of leader trustworthiness. *Pers. Rev.* 49 571–596. 10.1108/PR-02-2018-0054

[B39] Escribá-Carda N. Balbastre-Benavent F. Canet-Giner M. T. (2017). Employees’ perceptions of high-performance work systems and innovative behaviour: the role of exploratory learning. *Eur. Manage. J.* 35 273–281. 10.1016/j.emj.2016.11.002

[B40] Fang Y. Qureshi I. Sun H. McCole P. Ramsey E. Lim K. H. (2014). Trust, satisfaction, and online repurchase intention. *MIS Q.* 38 407–A409.

[B41] Fang Y.-C. Chen J.-Y. Wang M.-J. Chen C.-Y. (2019). The impact of inclusive leadership on employees’ innovative behaviors: the mediation of psychological capital. *Front. Psychol.* 10:1803. 10.3389/fpsyg.2019.01803 31447740 PMC6691172

[B42] Fang Y.-C. Ren Y.-H. Chen J.-Y. Chin T. Yuan Q. Lin C.-L. (2021). Inclusive leadership and career sustainability: mediating roles of supervisor developmental feedback and thriving at work. *Front. Psychol.* 12:671663. 10.3389/fpsyg.2021.671663 34295283 PMC8291222

[B43] Fornell C. Lacker D. (1981). Two structural equation models with unobservable variables and measurement error. *J. Mark. Res.* 18 39–50. 10.2307/3151312

[B44] Foss L. Woll K. Moilanen M. (2013). Creativity and implementations of new ideas: do organisational structure, work environment and gender matter? *Int. J. Gender Entrep*. 5 298–322. 10.1108/ijge-09-2012-0049

[B45] George J. M. Zhou J. (2007). Dual tuning in a supportive context: joint contributions of positive mood, negative mood, and supervisory behaviors to employee creativity. *Acad. Manage. J.* 50 605–622. 10.5465/amj.2007.25525934

[B46] Grant A. M. Berry J. W. (2011). The necessity of others is the mother of invention: intrinsic and prosocial motivations, perspective taking, and creativity. *Acad. Manage. J.* 54 73–96. 10.5465/amj.2011.59215085

[B47] Guan J. Liu N. (2016). Exploitative and exploratory innovations in knowledge network and collaboration network: a patent analysis in the technological field of nano-energy. *Res. Policy* 45 97–112. 10.1016/j.respol.2015.08.002

[B48] Gumusluoglu L. Ilsev A. (2009). Transformational leadership, creativity, and organizational innovation. *J. Bus. Res.* 62 461–473. 10.1016/j.jbusres.2007.07.032

[B49] Hair J. F. Hult G. T. M. Ringle C. M. Sarstedt M. (2013). *A Primer on Partial Least Squares Structural Equation Modeling (PLS-SEM).* Thousand Oaks, CA: Sage.

[B50] Han Y. Yang B.-Y. Zhang P.-C. (2011). Organizational commitment leads to employee innovative performance: a moderated effect of goal orientation. *Stud. Sci. Sci.* 29 127–137.

[B51] Hayes A. F. (2009). Beyond Baron and Kenny: statistical mediation analysis in the new millennium. *Commun. Monogr.* 76 408–420. 10.1080/03637750903310360

[B52] Henseler J. Ringle C. M. Sinkovics R. R. (2009). “The use of partial least squares path modeling in international marketing,” in *New Challenges to International Marketing*, eds Sinkovics R. R. Ghauri P. N. (Bingley: Emerald Group Publishing Limited). 10.2196/jmir.3122

[B53] Hirak R. Peng A. C. Carmeli A. Schaubroeck J. M. (2012). Linking leader inclusiveness to work unit performance: the importance of psychological safety and learning from failures. *Leadersh. Q.* 23 107–117. 10.1016/j.leaqua.2011.11.009

[B54] Hollander E. P. (2013). “Inclusive leadership and idiosyncrasy credit in leader-follower relations,” in *The Oxford Handbook of Leadership*, ed. Rumsey M. G. (Oxford: Oxford University Press), 122–143.

[B55] Hu Y. Zhu L. Zhou M. Li J. Maguire P. Sun H. (2018). Exploring the influence of ethical leadership on voice behavior: how leader-member exchange, psychological safety and psychological empowerment influence employees’ willingness to speak out. *Front. Psychol.* 9:1718. 10.3389/fpsyg.2018.01718 30258392 PMC6143838

[B56] Imran R. Anis-ul-Haque M. (2011). Mediating effect of organizational climate between transformational leadership and innovative work behaviour. *Pak. J. Psychol. Res.* 183–199.

[B57] Janssen O. (2000). Job demands, perceptions of effort-reward fairness and innovative work behaviour. *J. Occup. Organ. Psychol.* 73 287–302. 10.1348/096317900167038

[B58] Janssen O. Van Yperen N. W. (2004). Employees’ goal orientations, the quality of leader-member exchange, and the outcomes of job performance and job satisfaction. *Acad. Manage. J.* 47 368–384. 10.5465/20159587

[B59] Janssen S. Moeller K. Schlaefke M. (2011). Using performance measures conceptually in innovation control. *J. Manage. Control* 22:107. 10.1007/s00187-011-0130-y

[B60] Jaussi K. S. Dionne S. D. (2003). Leading for creativity: the role of unconventional leader behavior. *Leadersh. Q.* 14 475–498. 10.1016/s1048-9843(03)00048-1

[B61] Javed B. Abdullah I. Zaffar M. A. ul Haque A. Rubab U. (2019a). Inclusive leadership and innovative work behavior: the role of psychological empowerment. *J. Manage. Organ.* 25 554–571. 10.1186/s12913-020-05129-1 32228574 PMC7106698

[B62] Javed B. Khan A. A. Bashir S. Arjoon S. (2017). Impact of ethical leadership on creativity: the role of psychological empowerment. *Curr. Issues Tour.* 20 839–851. 10.1080/13683500.2016.1188894

[B63] Javed B. Khan A. K. Quratulain S. (2018). Inclusive leadership and innovative work behavior: examination of LMX perspective in small capitalized textile firms. *J. Psychol.* 152 594–612. 10.1080/00223980.2018.1489767 30260768

[B64] Javed B. Naqvi S. M. M. R. Khan A. K. Arjoon S. Tayyeb H. H. (2019b). Impact of inclusive leadership on innovative work behavior: the role of psychological safety. *J. Manage. Organ.* 25 117–136. 10.1017/jmo.2017.3

[B65] Jiang J. Chen C. Dai B. Shi G. Ding G. Liu L. (2015). Leader emergence through interpersonal neural synchronization. *Proc. Natl. Acad. Sci. U.S.A.* 112 4274–4279. 10.1073/pnas.1422930112 25831535 PMC4394311

[B66] Jin M. Lee J. Lee M. (2017). Does leadership matter in diversity management? Assessing the relative impact of diversity policy and inclusive leadership in the public sector. *Leadersh. Organ. Dev. J.* 38 303–319. 10.1108/lodj-07-2015-0151

[B67] Jose G. Mampilly S. R. (2014). Psychological empowerment as a predictor of employee engagement: an empirical attestation. *Glob. Bus. Rev.* 15 93–104. 10.1177/0972150913515589

[B68] Kang J. H. Solomon G. T. Choi D. Y. (2015). CEOs’ leadership styles and managers’ innovative behaviour: investigation of intervening effects in an entrepreneurial context. *J. Manage. Stud.* 52 531–554. 10.1111/joms.12125

[B69] Kaptein M. (2011). From inaction to external whistleblowing: the influence of the ethical culture of organizations on employee responses to observed wrongdoing. *J. Bus. Ethics* 98 513–530. 10.1007/s10551-010-0591-1

[B70] Ke W. Zhang P. (2008). “Participating in open source software projects: the role of empowerment,” in *Proceedings of the Pre-ICIS Workshop on HCI Research in MIS*, Paris.

[B71] Kent M. (2014). “Approach/engagement and withdrawal/defense as basic biobehavioral adaptations: resilient transcendence of a popular duality,” in *The Resilience Handbook: Approaches to Stress and Trauma*, eds Kent M. Davis M. C. Reich J. W. (London: Routledge), 33–43.

[B72] Kessel M. Hannemann-Weber H. Kratzer J. (2012). Innovative work behavior in healthcare: the benefit of operational guidelines in the treatment of rare diseases. *Health Policy* 105 146–153. 10.1016/j.healthpol.2012.02.010 22405486

[B73] Kline R. B. (2011). *Principles and Practice of Structural Equation Modeling*. New York, NY: Guilford Press.

[B74] Lapierre L. M. Hackett R. D. Taggar S. (2006). A test of the links between family interference with work, job enrichment and leader–member exchange. *Appl. Psychol.* 55 489–511. 10.1111/j.1464-0597.2006.00234.x

[B75] Lee Y. Tao W. Li J.-Y. Q. Sun R. (2020). Enhancing employees’ knowledge sharing through diversity-oriented leadership and strategic internal communication during the COVID-19 outbreak. *J. Knowl. Manage.* 25 1526–1549. 10.1108/jkm-06-2020-0483

[B76] Lee Y.-H. Hsieh Y.-C. Hsu C.-N. (2011). Adding innovation diffusion theory to the technology acceptance model: supporting employees’ intentions to use e-learning systems. *J. Educ. Technol. Soc.* 14 124–137.

[B77] Li A. McCauley K. D. Shaffer J. A. (2017). The influence of leadership behavior on employee work-family outcomes: a review and research agenda. *Hum. Resour. Manag. Rev.* 27 458–472. 10.1016/j.hrmr.2017.02.003

[B78] Liu D. Gong Y. Zhou J. Huang J.-C. (2017). Human resource systems, employee creativity, and firm innovation: the moderating role of firm ownership. *Acad. Manage. J.* 60 1164–1188. 10.5465/amj.2015.0230

[B79] Liu Y. Jing Y. Gao M. (2015). Transformational leadership: from the perspective of neurological leadership. *Open J. Leadersh.* 4 143–152. 10.4236/ojl.2015.44013

[B80] Luthans F. Youssef C. M. Avolio B. J. (2007). *Psychological Capital: Developing the Human Competitive Edge*, Vol. 198 Oxford: Oxford University Press Oxford.

[B81] Madjar N. Greenberg E. Chen Z. (2011). Factors for radical creativity, incremental creativity, and routine, noncreative performance. *J. Appl. Psychol.* 96 730–743. 10.1037/a0022416 21319879

[B82] Maslyn J. M. Schyns B. Farmer S. M. (2017). Attachment style and leader-member exchange: the role of effort to build high quality relationships. *Leadersh. Organ. Dev. J*. 38 450–462. 10.1108/lodj-01-2016-0023

[B83] Mathisen G. E. Einarsen S. Mykletun R. (2012). Creative leaders promote creative organizations. *Int. J. Manpow*. 33 367–382. 10.1108/01437721211243741

[B84] Mitchell R. Boyle B. Parker V. Giles M. Chiang V. Joyce P. (2015). Managing inclusiveness and diversity in teams: how leader inclusiveness affects performance through status and team identity. *Hum. Resour. Manage.* 54 217–239. 10.1002/hrm.21658

[B85] Montag T. Maertz C. P. Jr. Baer M. (2012). A critical analysis of the workplace creativity criterion space. *J. Manage.* 38 1362–1386. 10.1177/0149206312441835

[B86] Montani F. Odoardi C. Battistelli A. (2014). Individual and contextual determinants of innovative work behaviour: proactive goal generation matters. *J. Occup. Organ. Psychol.* 87 645–670. 10.1111/joop.12066

[B87] Mumford M. D. Scott G. M. Gaddis B. Strange J. M. (2002). Leading creative people: orchestrating expertise and relationships. *Leadersh. Q.* 13 705–750. 10.1016/s1048-9843(02)00158-3

[B88] Mumtaz S. Parahoo S. K. (2019). Promoting employee innovation performance: examining the role of self-efficacy and growth need strength. *Int. J. Product. Perform. Manage*. 69 704–722. 10.1108/ijppm-12-2017-0330

[B89] Nauman S. Khan A. M. Ehsan N. (2010). Patterns of empowerment and leadership style in project environment. *Int. J. Proj. Manage.* 28 638–649. 10.1111/j.1365-2834.2008.00930.x 18808470

[B90] Nembhard I. M. Edmondson A. C. (2006). Making it safe: the effects of leader inclusiveness and professional status on psychological safety and improvement efforts in health care teams. *J. Organ. Behav.* 27 941–966. 10.1002/job.413

[B91] Nishii L. H. Mayer D. M. (2009). Do inclusive leaders help to reduce turnover in diverse groups? The moderating role of leader–member exchange in the diversity to turnover relationship. *J. Appl. Psychol.* 94 1412–1426. 10.1037/a0017190 19916652

[B92] Orth M. Volmer J. (2017). Daily within-person effects of job autonomy and work engagement on innovative behaviour: the cross-level moderating role of creative self-efficacy. *Eur. J. Work Organ. Psychol.* 26 601–612. 10.1080/1359432x.2017.1332042

[B93] Pardo-del-Val M. Martínez-Fuentes C. Roig-Dobón S. (2012). Participative management and its influence on organizational change. *Manage. Decis.* 50 1843–1860. 10.1108/00251741211279639

[B94] Prihatiningsih W. (2016). Pengaruh Perceived Organizational Support dan Psychological Empowerment Terhadap Organizational Citizenship Behaviour di Hotel Aston Cekareng. SKRIPSI-2015. Available online at: http://repository.trisakti.ac.id/usaktiana/index.php/home/detail/detail_koleksi/0/SKR/judul/00000000000000082143/0 (accessed August 17, 2021).

[B95] Pundt A. (2015). The relationship between humorous leadership and innovative behavior. *J. Manag. Psychol*. 30 878–893. 10.3389/fpsyg.2019.01636 31379671 PMC6659772

[B96] Qi L. Liu B. Wei X. Hu Y. (2019). Impact of inclusive leadership on employee innovative behavior: perceived organizational support as a mediator. *PLoS One* 14:e0212091. 10.1371/journal.pone.0212091 30817753 PMC6395022

[B97] Qu R. Janssen O. Shi K. (2017). Leader–member exchange and follower creativity: the moderating roles of leader and follower expectations for creativity. *Int. J. Hum. Resour. Manage.* 28 603–626. 10.1080/09585192.2015.1105843

[B98] Rahman S. Batool S. Akhtar N. Ali H. (2015). Fostering individual creativity through proactive personality: a multilevel perspective. *J. Manag. Sci.* 9:2.

[B99] Ramamoorthy N. Flood P. C. Slattery T. Sardessai R. (2005). Determinants of innovative work behaviour: development and test of an integrated model. *Creat. Innov. Manage.* 14 142–150. 10.1111/j.1467-8691.2005.00334.x

[B100] Randel A. E. Galvin B. M. Shore L. M. Ehrhart K. H. Chung B. G. Dean M. A. (2018). Inclusive leadership: realizing positive outcomes through belongingness and being valued for uniqueness. *Hum. Resour. Manage. Rev.* 28 190–203. 10.1016/j.hrmr.2017.07.002

[B101] Raoof R. Basheer M. F. Javeria S. Ghulam Hassan S. Jabeen S. (2021). Enterprise resource planning, entrepreneurial orientation, and the performance of SMEs in a South Asian economy: the mediating role of organizational excellence. *Cogent Bus. Manage.* 8:1973236.

[B102] Rawat P. S. Lyndon S. Pradhan M. R. Jose J. Kollenchira M. Mehta G. (2020). Employee reactiveness and inclusive leadership: time to manage emotional diversity. *South Asian J. Bus. Stud.* 10 357–376. 10.1108/sajbs-02-2020-0042

[B103] Reiter-Palmon R. Illies J. J. (2004). Leadership and creativity: understanding leadership from a creative problem-solving perspective. *Leadersh. Q.* 15 55–77. 10.1016/j.leaqua.2003.12.005

[B104] Riggle R. J. Edmondson D. R. Hansen J. D. (2009). A meta-analysis of the relationship between perceived organizational support and job outcomes: 20 years of research. *J. Bus. Res.* 62 1027–1030. 10.1016/j.jbusres.2008.05.003

[B105] Ringle C. Wende S. Becker J. (2015). *SmartPLS 3 [Computer Software].* Boenningstedt: SmartPLS GmbH.

[B106] Rowlinson S. Cheung Y. K. F. (2008). Stakeholder management through empowerment: modelling project success. *Constr. Manage. Econ.* 26 611–623. 10.1080/01446190802071182

[B107] Ryan M. K. Haslam S. A. (2007). The glass cliff: exploring the dynamics surrounding the appointment of women to precarious leadership positions. *Acad. Manage. Rev.* 32 549–572. 10.5465/amr.2007.24351856

[B108] Ryan R. M. Deci E. L. (2000a). Intrinsic and extrinsic motivations: classic definitions and new directions. *Contemp. Educ. Psychol.* 25 54–67. 10.1006/ceps.1999.1020 10620381

[B109] Ryan R. M. Deci E. L. (2000b). Self-determination theory and the facilitation of intrinsic motivation, social development, and well-being. *Am. Psychol.* 55 68–78. 10.1037//0003-066x.55.1.68 11392867

[B110] Sabir S. A. Mohammad H. B. Shahar H. B. K. (2019). The role of overconfidence and past investment experience in herding behaviour with a moderating effect of financial literacy: evidence from Pakistan stock exchange. *Asian Econ. Financ. Rev.* 9 480–490. 10.18488/journal.aefr.2019.94.480.490

[B111] Schermuly C. C. Büsch V. Graßmann C. (2017). Psychological empowerment, psychological and physical strain and the desired retirement age. *Pers. Rev.* 46, 950–969. 10.1108/pr-06-2015-0159

[B112] Schermuly C. C. Meyer B. Dämmer L. (2013). Leader-member exchange and innovative behavior. *J. Pers. Psychol.* 11:182. 10.3390/bs11120182 34940117 PMC8698413

[B113] Scott S. G. Bruce R. A. (1994b). Determinants of innovative behavior: a path model of individual innovation in the workplace. *Acad. Manage. J.* 37 580–607. 10.5465/256701

[B114] Scott S. G. Bruce R. A. (1994a). “Creating innovative behavior among R&D professionals: the moderating effect of leadership on the relationship between problem-solving style and innovation,” in *Proceedings of 1994 IEEE International Engineering Management Conference-IEMC’94*, Dayton North, OH.

[B115] Seibert S. E. Wang G. Courtright S. H. (2011). Antecedents and consequences of psychological and team empowerment in organizations: a meta-analytic review. *J. Appl. Psychol.* 96 981–1003. 10.1037/a0022676 21443317

[B116] Sekaran U. Bougie R. (2003). *Research Methods for Business: A Skill Building Approach.* Singapore: Willey.

[B117] Shalley C. E. Gilson L. L. Blum T. C. (2009). Interactive effects of growth need strength, work context, and job complexity on self-reported creative performance. *Acad. Manage. J.* 52 489–505. 10.5465/amj.2009.41330806

[B118] Sharifirad M. S. (2013). Transformational leadership, innovative work behavior, and employee well-being. *Glob. Bus. Perspect.* 1 198–225. 10.1007/s40196-013-0019-2

[B119] Shin S. J. Yuan F. Zhou J. (2017). When perceived innovation job requirement increases employee innovative behavior: a sensemaking perspective. *J. Organ. Behav.* 38 68–86. 10.1002/job.2111

[B120] Shin S. J. Zhou J. (2003). Transformational leadership, conservation, and creativity: evidence from Korea. *Acad. Manage. J.* 46 703–714. 10.2307/30040662

[B121] Shore L. M. Clevel J. N. Sanchez D. (2018). Inclusive workplaces: a review and model. *Hum. Resour. Manage. Rev.* 28 176–189. 10.1016/j.hrmr.2017.07.003

[B122] Simsek Z. Jansen J. J. Minichilli A. Escriba-Esteve A. (2015). Strategic leadership and leaders in entrepreneurial contexts: a nexus for innovation and impact missed? *J. Manage. Stud.* 52 463–478. 10.1111/joms.12134

[B123] Singh K. (2007). *Quantitative Social Research Methods*. Thousand Oaks, CA: Sage.

[B124] Song W. Ming X. Han Y. Xu Z. Wu Z. (2015). An integrative framework for innovation management of product–service system. *Int. J. Prod. Res.* 53 2252–2268. 10.1080/00207543.2014.932929

[B125] Spreitzer G. M. (1995). Psychological empowerment in the workplace: dimensions, measurement, and validation. *Acad. Manage. J.* 38 1442–1465. 10.1111/jonm.12045 23815636

[B126] Spreitzer G. M. Kizilos M. A. Nason S. W. (1997). A dimensional analysis of the relationship between psychological empowerment and effectiveness satisfaction, and strain. *J. Managem.* 23 679–704. 10.1016/s0149-2063(97)90021-0

[B127] To M. L. Herman H. Ashkanasy N. M. (2015). A multilevel model of transformational leadership, affect, and creative process behavior in work teams. *Leadersh. Q.* 26 543–556. 10.1016/j.leaqua.2015.05.005

[B128] Tuuli M. M. Rowlinson S. (2009). Performance consequences of psychological empowerment. *J. Constr. Eng. Manage.* 135 1334–1347. 10.1061/(asce)co.1943-7862.0000103

[B129] Tuuli M. M. Rowlinson S. Fellows R. Liu A. M. (2015). Individual-level antecedents of psychological empowerment. *J. Manage. Eng.* 31:04014036. 10.1061/(asce)me.1943-5479.0000239

[B130] Wagner J. I. Cummings G. Smith D. L. Olson J. Anderson L. Warren S. (2010). The relationship between structural empowerment and psychological empowerment for nurses: a systematic review. *J. Nurs. Manage.* 18 448–462. 10.1111/j.1365-2834.2010.01088.x 20609049

[B131] Wang P. Rode J. C. (2010). Transformational leadership and follower creativity: the moderating effects of identification with leader and organizational climate. *Hum. Relat.* 63 1105–1128. 10.1177/0018726709354132

[B132] Wang P. Zhu W. (2011). Mediating role of creative identity in the influence of transformational leadership on creativity: Is there a multilevel effect? *J. Leadersh. Organ. Stud.* 18 25–39. 10.1177/1548051810368549

[B133] Wang S. Tomlinson E. C. Noe R. A. (2010). The role of mentor trust and protege internal locus of control in formal mentoring relationships. *J. Appl. Psychol.* 95 358–367. 10.1037/a0017663 20230075

[B134] Woodman R. W. Sawyer J. E. Griffin R. W. (1993). Toward a theory of organizational creativity. *Acad. Manage. Rev.* 18 293–321. 10.5465/amr.1993.3997517

[B135] Xiang H. Chen Y. Zhao F. (2017). “Inclusive leadership, psychological capital, and employee innovation performance: the moderating role of leader-member exchange,” in *Proceedings of the DEStech Transactions on Social Science, 2017 2nd International Conference on Humanities Science, Management and Education Technology (HSMET 2017), Education and Human Science*, Zhuhai.

[B136] Yan R. Basheer M. F. Irfan M. Rana T. N. (2020). Role of psychological factors in employee well-being and employee performance: an empirical evidence from Pakistan. *Rev. Argent. Clín. Psicol.* 29:638.

[B137] Yang J. Chang M. Chen Z. Zhou L. Zhang J. (2020). The chain mediation effect of spiritual leadership on employees’ innovative behavior. *Leadersh. Organ. Dev. J.* 42 114–129. 10.1108/lodj-10-2019-0442

[B138] Yang S.-Y. (2011). Wisdom displayed through leadership: exploring leadership-related wisdom. *Leadersh. Q.* 22 616–632. 10.1016/j.leaqua.2011.05.004

[B139] Yi H. (2008). The relationship between job performance and job satisfaction, organizational commitment, and goal orientation. *Acta Psychol. Sin.* 40:84. 10.1111/j.1365-2702.2010.03672.x 21492282

[B140] Yidong T. Xinxin L. (2013). How ethical leadership influence employees’ innovative work behavior: a perspective of intrinsic motivation. *J. Bus. Ethics* 116 441–455. 10.1007/s10551-012-1455-7

[B141] Yuan F. Woodman R. W. (2010). Innovative behavior in the workplace: the role of performance and image outcome expectations. *Acad. Manage. J.* 53 323–342. 10.5465/amj.2010.49388995

[B142] Yuanyuan Y. Y. H. (2013). Construct and measurement of knowledge Staff’s innovation performance. *Chin. J. Manage.* 1, 97–102.

[B143] Zafar M. Kousar S. Sabir S. A. Sajjad A. (2021). An exploratory study on academic, social and psychological problems faced by overseas students of higher education institutions of Pakistan. *J. Behav. Sci.* 31 46–69.

[B144] Zhou J. George J. M. (2001). When job dissatisfaction leads to creativity: Encouraging the expression of voice. *Acad. Manage. J.* 44 682–696. 10.5465/3069410

[B145] Zhu J. Xu S. Zhang B. (2020). The paradoxical effect of inclusive leadership on subordinates’ creativity. *Front. Psychol.* 10:2960. 10.3389/fpsyg.2019.02960 32038369 PMC6988563

